# Field-Cage Methodology for Evaluating Climatic Suitability for Introduced Wood-Borer Parasitoids: Preliminary Results from the Emerald Ash Borer System

**DOI:** 10.1673/031.011.14101

**Published:** 2011-10-31

**Authors:** Michael D. Ulyshen, Jian J. Duan, Leah S. Bauer, Juli Gould, Phil Taylor, Dick Bean, Carol Holko, Roy Van Driesche

**Affiliations:** ^1^ Department of Entomology, Michigan State University, East Lansing, MI 48824; ^2^ Current address: USDA Forest Service, Southern Research Station, Starkville, MS 39759; ^3^ USDA ARS, Beneficial Insects Introduction Research Unit, Newark, DE 19713; ^4^ USDA Forest Service, Northern Research Station, East Lansing, MI 48823; ^5^ USDA APHIS-PPQ, Buzzards Bay, MA 02542; ^6^ Maryland Department of Agriculture, Annapolis, MD 21401; ^7^ Department of Plant, Soil, and Insect Science, University of Massachusetts, Amherst, MA 01003

**Keywords:** classical biological control, emerald ash borer, exotic, *Fraxinus*, invasive, natural enemies

## Abstract

Field-cage methods were developed to evaluate the abilities of *Tetrastichus planipennisi* Yang (Hymenoptera: Eulophidae) and *Spathius agrili* Yang (Hymenoptera: Braconidae), biocontrol agents of *Agrilus planipennis* Fairmaire (Coleoptera: Buprestidae), to parasitize, develop and overwinter following three late-season releases at both a northern (Michigan) and a southern (Maryland) location within the current North American range of *A. planipennis.* In August, September and October of 2009, five young green ash trees were selected at each location. *Tetrastichus planipennisi* and *S. agrili* were each randomly assigned to one of two cages attached to each tree, surrounding separate sections of trunk in which late-instar *A. planipennis* had been inserted. The following April, the caged trunk sections were dissected to determine the fate of each *A. planipennis* larva and the developmental stages of all recovered parasitoid progeny. At both locations, *T. planipennisi* and *S. agrili* were able to parasitize hosts and successfully overwinter (i.e., reach adulthood the following spring). For *T. planipennisi*, successful parasitism (i.e., parasitoid progeny reached adulthood) occurred for all caged releases in Maryland, but only for the August and September releases in Michigan. At both locations, percent parasitism by *T. planipennisi* was higher in August and September than in October. For *S. agrili*, successful parasitism occurred for all caged releases in Maryland, but only for the August release in Michigan. In Maryland, percent parasitism by *S. agrili* in August and September was higher than in October. The caging method described here should be useful in determining the climatic suitability of other regions before proceeding with large-scale releases of either species and may have utility in other wood-borer parasitoid systems as well.

## Introduction

Although less than a decade has passed since the emerald ash borer, *Agrilus planipennis* Fairmaire (Coleoptera: Buprestidae), an invasive beetle from Asia, was first identified as the cause of ash tree (*Fraxinus* spp.) mortality in North America (Haack et al. 2002), the species has already become one of the most ecologically and economically significant invasive forest pests in North America (Gandhi and Herms 2009; Kovacs et al. 2010). In Michigan alone, *A. planipennis* has killed an estimated 40 million ash trees, with tens of millions more killed in other infested states and provinces (Emerald Ash Borer Information 2009). Efforts by regulatory agencies to eradicate *A. planipennis* were unsuccessful, and chemical control options are expensive (Sadof 2010), temporary, and not suited for widespread use (Poland and McCullough 2006; Herms et al. 2009). Although woodpeckers (Lindell et al. 2008) and some native parasitoid species (Cappaert and McCullough 2009) are important natural enemies of *A. planipennis* in North America, their overall impact is insufficient to reduce *A. planipennis* population densities below a lethal threshold for most ash trees (Bauer et al. 2004; Duan et al. 2009; Kula et al. 2010). A classical biological control program, involving three Hymenopteran parasitoids of *A. planipennis* in China (Liu et al. 2003, 2007; Gould et al. 2005), is under way in several infested states. The two larval parasitoids, *Tetrastichus planipennisi* Yang (Eulophidae) and *Spathius agrili* Yang (Braconidae), have so far been released in Michigan, Maryland, Ohio, and Illinois.

*Tetrastichus planipennisi* is a gregarious koinobiont endoparasitoid that preferentially parasitizes large larvae (Liu et al. 2003; Yang et al. 2006; Liu and Bauer 2007; Ulyshen et al. 2010b; Ulyshen et al. in press). Adults range from 1.6 to 4.1 mm in length with a female to male sex ratio of ∼ 2.5:1 (Yang et al. 2006). *Agrilus planipennis* larvae parasitized by *T. planipennisi* remain active and continue to feed for about a week. Between 4 and 172 offspring are produced per host (Ulyshen et al. 2010a). After consuming the host larva, parasitoid larvae exit from the integument and pupate within the *A. planipennis* gallery (Yang et al. 2006). In China, four or more generations are produced per year and levels of parasitism average 22.4% (Liu et al. 2007), ranging from 0% to 65% (Liu et al. 2003; Liu et al. 2007). Although *T. planipennisi* overwinters as larvae or pupae in a dormant state (pers. obs.; Liu et al. 2007), there is no evidence that the species enters diapause.

*Spathius agrili* is a gregarious idiobiont ectoparasitoid that also prefers to parasitize older *A. planipennis* larvae (Yang et al. 2005). Adults range from 3.4 to 4.3 mm in length with a female to male sex ratio of ∼ 3:1 (Yang et al. 2005). Females permanently paralyze their hosts by envenomation when ovipositing and produce 1–20 offspring per host (Yang et al. 2005, 2010). After consuming the host larva, mature larvae spin individual cocoons within the gallery (Yang et al. 2005). Adults from a single brood exit from their cocoons and emerge from the tree over a period of 5 to 44 days (Yang et al. 2010). In China, the species completes up to four generations per year and levels of parasitism range from 30% to 90% (Yang et al. 2005, 2010). *Spathius agrili* overwinters as prepupae inside reinforced cocoons (Yang et al. 2010) and, unlike *T. planipennisi*, apparently enters diapause (JG, unpublished data), presumably in response to changing photoperiod and/or temperature (Leather et al. 1993).

Whereas rearing methods have been developed for both species and releases began in 2007 (Bauer et al. 2008; Ulyshen et al. 2010a; Yang et al. 2010), information on where and when to release either species is lacking. This is problematic considering that, historically, most introduced biological control agents have failed to be established (Clausen 1956) and the inability to adapt to the new climate has been responsible for more of these failures than any other single factor (DeBach and Bartlett 1964). In addition, because natural enemies are usually restricted to narrower geographic or ecological ranges than their hosts (Messenger and van den Bosch 1971), it may not be realistic to expect *T. planipennisi* and *S. agrili* to establish and persist wherever *A. planipennis* occurs in North America. Although the distributions of *T. planipennisi* and *S. agrili* are known to overlap in China (Liu et al. 2003), the extent to which this is the case, and whether their ranges fully encompass the distribution of the emerald ash borer in Asia, remain unclear.

Clearly, large-scale releases of these species will be more effective where and when conditions have been shown to be suitable. The ability to successfully overwinter, for example, should be demonstrated prior to proceeding with any large-scale releases (Leather et al. 1993), but this ability has not been well-documented for either species anywhere in North America. In addition, the point at which it becomes too late in the season to expect successful parasitism and establishment following a parasitoid release is likely to vary between species and with latitude, but this too remains unexplored. A major obstacle in addressing these questions has been the lack of a reliable sampling method for evaluating the establishment of wood-borer parasitoids.

In the present study, field-cage methods were developed to preliminarily test the abilities of *T. planipennisi* and *S. agrili* to parasitize, develop and overwinter following three late-season releases at both a northern (Michigan) and a southern (Maryland) location within the current North American range of *A. planipennis*. In addition to assessing the utility of the caging method, the information gained from this study should help determine where and when these species should be released to best achieve establishment and, ultimately, control of *A. planipennis*.

## Materials and Methods

**Study Sites.** This research took place simultaneously at two locations in the U.S. The first was a second-growth wetland forest (i.e., seasonally flooded) bordering a small stream in Ingham County, Michigan (42° 43' 98″ N, 84° 25′ 38″ W). In addition to green ash, *Fraxinus pennsylvanica* Marshall (Lamiales: Oleaceae), other tree species common at the site included boxelder, *Acer negundo* L.(Sapindales: Sapindaceae), burroak, *Quercus macrocarpa* Michaux (Fagales: Fagaceae), black cherry, *Prunus serotina* Ehrhart (Rosales: Rosaceae), red maple, *Acer rubrum* L. (Sapindales: Sapindaceae) and cottonwood, *Populus deltoides* Bartram Ex Marshall (Malpighiales: Salicaceae). The second location was a second-growth wetland forest with poorly drained soils dominated by *F. pennsylvanica* and *A. rubrum* in Prince George's County, MD (38° 42′ 31″ N, 76° 53′95″ W). On average, the Michigan site is approximately five to seven degrees Celsius cooler than the Maryland site throughout the year and gets nearly 20 cm less rain annually (rssweather.com, accessed 3/30/2010).

**Parasitoids.** Laboratory colonies of *T. planipennisi*, originally collected in China (Liaoning province), and *S. agrili*, also originally collected in China (Tianjin city), were used in this study. Only naïve wasps that had not been presented with hosts were used in experiments. They were at least one week old at the time of use and were presumed to have mated because mating is known to occur almost immediately for both species following female emergence (personal observation).

**Field cages.** On three occasions in the fall of 2009 (25–26 August, 21–22 September and 13 October), five young green ash trees 4–7 cm in diameter at 1.5 m were selected at each location (i.e., 15 trees per location, 30 trees in total). Two cages were attached to each tree, surrounding separate ∼ 0.5 m sections of trunk, in which ten third- or fourth-instar, field-collected *A. planipennis* had been inserted. The heights of the cages varied somewhat among trees, but never exceeded 3 m. The larvae were inserted into narrow grooves chiseled beneath small flaps of bark. The larvae were inserted “head down” to encourage feeding and the flaps of bark were held closed over the larvae with thin strips of Parafilm. The ten larvae in each trunk section were arranged in two “rings” encircling the trunk, each with 5 larvae, with the rings being ∼ 10 cm apart. All larvae were inserted 1–3 m above the ground. The cages were made of fine fabric screening held ∼ 5–10 cm away from the trunk by metal wire frames attached to each tree. Water in a vial with a cotton wick and honey streaked on the inside surface of a Petri dish lid were suspended with string in each cage. A section of plastic sheeting was stapled to the tree above each cage to provide shelter during rainstorms. For pictures of similar cages, see Ulyshen et al. (2010a). The cages were constructed over a two-day period; *A. planipennis* larvae were inserted and the wire frames attached on the first day and water, honey, screening and wasps were added on the following day. Staples were used to connect the edges of the screen and rubber bands were used to secure the screen to the trunk at the top and bottom of the cage. For each tree, 10 ♀ and ∼ 5 ♂ *T. planipennisi* were randomly assigned to one cage and 5 ♀ and ∼ 5 ♂ *S. agrili* to the other.

The following April, the caged trunk sections were dissected to assign each *A. planipennis* larva to one of the five following fates: parasitized (by *T. planipennisi* or *S. agrili*), preyed upon (including parasitism by native parasitoids), alive, dead (not killed by a predator or parasitoid), or lost (larvae that could not be found at the time of sampling). At the time of sampling, *T. planipennisi* progeny were assigned to one of the following four categories: larva, pupa, emerged adult (still in the galleries) and exited adult (already emerged from the tree as evidenced by emergence holes through the bark). Because all *S. agrili* progeny at the time of sampling were inside cocoons, their precise stages of development could not be determined. All recovered parasitoid broods were individually placed in Falcon Petri dishes (50 × 9 mm with tight-fit lid) lined with moistened filter paper and reared in the laboratory to determine overwintering survival.

**Data Analysis.** Because larvae were inserted into the ash trunks by different groups of people in Michigan and in Maryland, and ash tree health varied between locations (i.e., the trees selected in Michigan generally appeared healthier than those used in Maryland), data on parasitism and other fates of *A. planipennis* larvae for each parasitoid species at each location were analyzed separately with the likelihood Chi-square test (SAS Institute 2008). Percent parasitism calculations were based on all *A. planipennis* larvae recovered at the end of the experiment, including dead larvae as these were all healthy and actively feeding when inserted into the trees (Note: although some larvae may have died soon after insertion, making them essentially unavailable for parasitism, the extent to which this was the case cannot be determined). All of the *A. planipennis* larvae that were lost due to insufficient tightening of the bark flaps or destroyed during the sampling process, however, were excluded from the data set.

## Results

**Figure 1.**
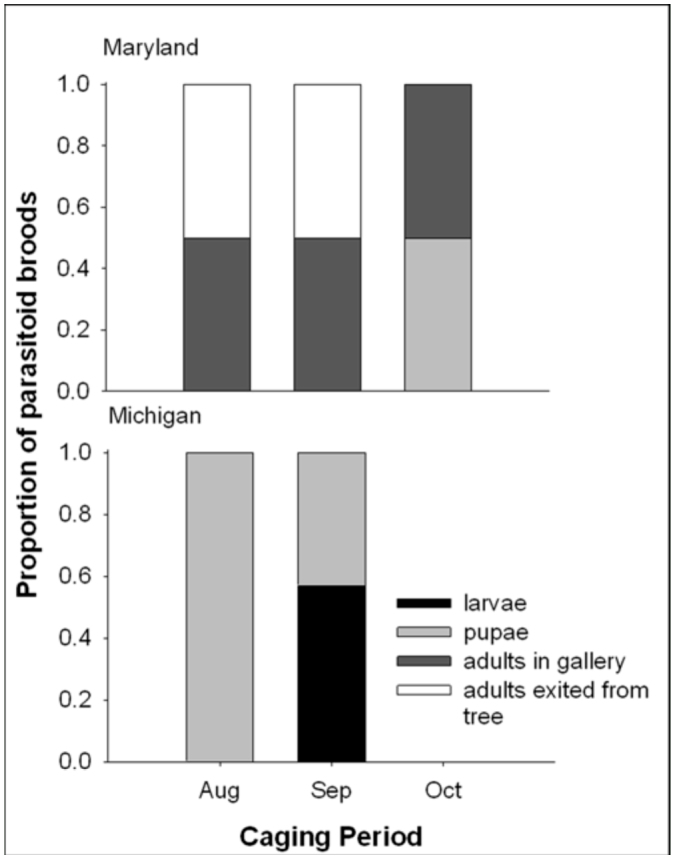
Developmental stages of *T. planipennisi* at the time of sampling. High quality figures are available online.

At both locations, *T. planipennisi* and *S. agrili* were able to parasitize hosts and overwinter at advanced stages of development. *Tetrastichus planipennisi* overwintered as late-instars or pupae after exiting the host, with about 89% (N=614) and 98% (N=208) of the progeny recovered from Michigan and Maryland, respectively, reaching adulthood in the laboratory. *Spathius agrili* overwintered inside cocoons, with about 53% (N=12) and 50% (N=36) of the progeny recovered from Michigan and Maryland, respectively, reaching adulthood in the laboratory. *T. planipennisi* progeny were more developed at the time of sampling in Maryland than in Michigan due to climatic differences between the two locations (Figure 1). For example, all *T. planipennisi* broods from the first two caging periods in Maryland were adults at the time of sampling and half of the adults had already exited the trees. In Michigan, by contrast, all *T. planipennisi* broods from the first caging period consisted of pupae and those from the second caging period consisted of pupae as well as larvae. While *S. agrili* progeny were likely at more advanced stages of development in Maryland as well, all progeny recovered at both locations were within cocoons so their precise stages of development could not be determined.

**Figure 2.**
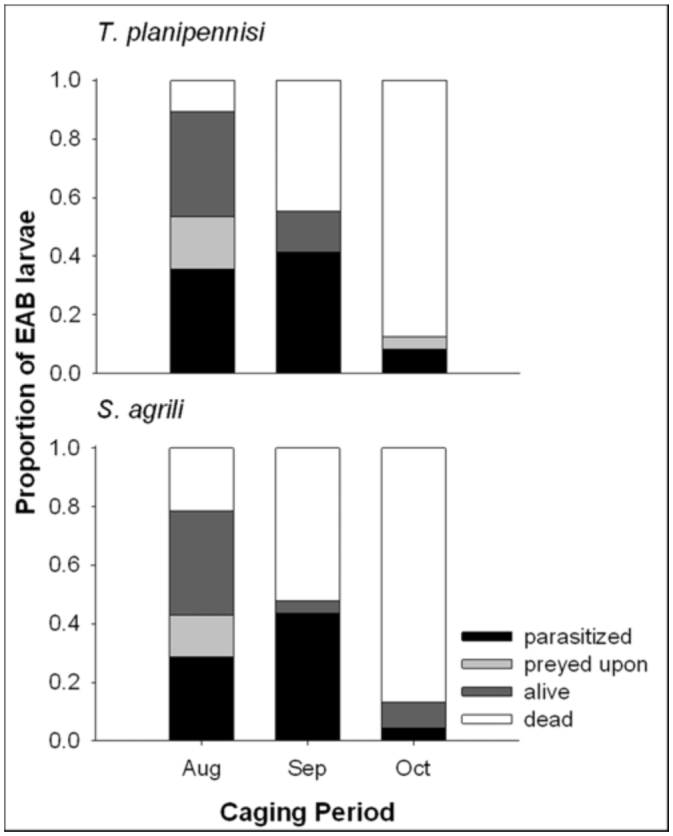
Fates of emerald ash borer larvae exposed to *T. planipennisi* or *S. agrili* at three times during the fall of 2009 in Maryland. High quality figures are available online.

**Figure 3.**
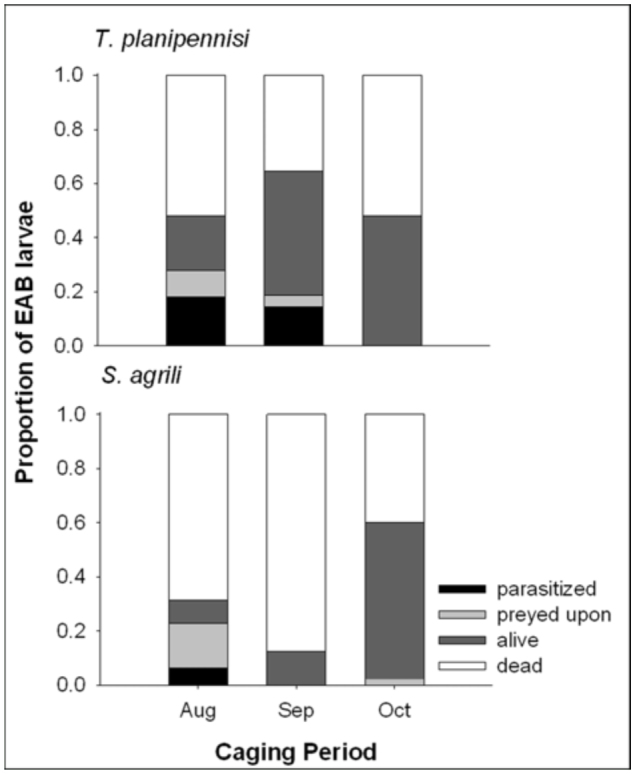
Fates of emerald ash borer larvae exposed to *T. planipennisi* or *S. agrili* at three times during the fall of 2009 in Michigan. High quality figures are available online.

In both Maryland (Figure 2) and Michigan (Figure 3), caging period significantly affected percent parasitism of *A. planipennis* hosts exposed to *T. planipennisi* (Maryland: N = 81, χ^2^ = 45.147, df = 6, *p* < 0.0001; Michigan: N = 146, χ^2^ = 28.759, df = 6, P<0.0001) or *S. agrili* (Maryland: N = 60; χ^2^ = 27.643, df = 6, *p* < 0.0001; Michigan: N = 141, χ^2^ = 53.124, df = 6, *p* < 0.0001), although this may be due in part to the fact that the mortality rate of *A. planipennis* larvae varied considerably among caging periods, particularly in Maryland (Figures 2 and 3). For *T. planipennisi*, successful parasitism occurred during all three caging periods in Maryland (Figure 2), but only during the August and September periods in Michigan (Figure 3). Percent parasitism by *T. planipennisi* in August (18.0 and 35.7%) and September (14.6 and 41.4%) was higher than in October (0.0 and 8.3%) in both Michigan and Maryland, respectively. Similarly, in Maryland, percent parasitism by *S. agrili* in August (28.6%) and September (43.48%) was significantly higher than in October (4.4%). In Michigan, by contrast, only 6.25% parasitism by *S. agrili* was observed in August and no parasitism was observed in September or October.

## Discussion

At both locations, caging period significantly affected the parasitism rate and stage of development of *T. planipennisi* and *S. agrili.* Parasitism was recorded for both species from each caging period in Maryland, but at considerably reduced rates in October. It should be noted, however, that, for reasons unknown, a large proportion of the *A. planipennis* larvae inserted in October in Maryland were dead at the time of sampling. Although the extent to which this was the case cannot be determined, it is possible that some mortality took place soon enough after insertion to affect observed parasitism rates. In Michigan, parasitism was not recorded for either species in October and was recorded only for *T. planipennisi* in September. Though not detectable at the time of sampling, it is possible that some parasitism occurred following these releases but the parasitoid progeny were unable to develop adequately within or upon the host before the onset of winter. Alternatively, the apparent lack of parasitism in October in Michigan may be attributable to cool temperatures on the morning the wasps were added to the cages given that current weather conditions, especially temperature, can determine whether a field release will succeed or fail (Stiling 1993; Etzel and Legner 1999). Regardless, the results from this study suggest late-season releases of either species should be avoided when possible.

The parasitism rates exhibited by *T. planipennisi* and *S. agrili* were similar in Maryland for the three caging periods. In Michigan, by contrast, a greater proportion of hosts were parasitized by *T. planipennisi* than *S. agrili* in August and parasitism was recorded only by *T. planipennisi* in September. That *S. agrili* parasitized at lower rates than *T. planipennisi* in Michigan may be due, in part, to the fact that greater proportions of *A. planipennis* larvae died in August and September in the *S. agrili* cages (Figure 3). It remains unknown, however, if this mortality occurred soon enough after insertion to affect observed parasitism rates. In a field-cage study carried out within the same Michigan forest but several months earlier (i.e., June and July 2009), the number of hosts parasitized by *S. agrili* was comparable to that parasitized by *T. planipennisi* (Ulyshen et al. 2010a). (It should be noted that in the previous study, three female *S. agrili* or 10 female *T. planipennisi* were added to each cage compared to the five or 10, respectively, used in the present study). These inconsistent findings may either suggest that *A. planipennis* mortality did occur early enough in the present study to affect observed parasitism rates or that the effectiveness of *S. agrili* declines more rapidly near the end of the season than does that of *T. planipennisi.* More research will be needed to resolve this issue.

The caging methods described in this study should prove useful in assessing the climatic suitability of other sites before proceeding with large-scale releases of either species and may have utility in other wood-borer parasitoid systems as well. It is important to note, however, that a single documentation of overwintering following a field release is not conclusive evidence of suitability as it is common in biological control to document parasitism at immediate release sites even when conditions there are not suitable to maintain permanent populations (DeBach and Bartlett 1964). Consequently, long-term monitoring of release sites will be needed in order to fully assess the potential ranges and impacts of *T. planipennisi* and *S. agrili* in North America.
